# Cyclin Y interacts with Chk1 to activate RRM2/STAT3 signaling and promotes radioresistance in non-small cell lung cancer

**DOI:** 10.7150/ijbs.106925

**Published:** 2025-02-18

**Authors:** Zhiwei Liu, Huichan Xue, Zhi Wang, Ye Zhao, Shuangbing Xu, Xiaorong Dong

**Affiliations:** 1 Cancer Center, Union Hospital, Tongji Medical College, Huazhong University of Science and Technology, Wuhan 430022, China.; 2 Hubei Key Laboratory of Precision Radiation Oncology, Wuhan 430022, China.; 3 Institute of Radiation Oncology, Union Hospital, Tongji Medical College, Huazhong University of Science and Technology, Wuhan 430022, China.

**Keywords:** cyclin Y, Chk1, RRM2, radioresistance, non-small cell lung cancer

## Abstract

Radioresistance is one of the main reasons for the recurrence and metastasis of non-small cell lung cancer. Cyclin Y has been implicated in various cellular processes such as cell growth, proliferation, autophagy, and tumor progression. However, the function and regulatory mechanism of Cyclin Y in lung cancer radioresistance remain poorly understood. In this study, we find that Cyclin Y is overexpressed in non-small cell lung cancer and correlates with poor prognosis. Furthermore, knockdown of Cyclin Y results in inhibited cell growth and proliferation, increases DNA damage, impairs DNA damage repair, and enhances radiosensitivity *in vitro* and* in vivo*. Mechanistically, we uncover that Cyclin Y interacts with Chk1 and positively regulate both the mRNA and protein levels of RRM2, resulting in increased STAT3 phosphorylation. Rescue experiments confirm that the effects of Cyclin Y on lung cancer are mediated partially by RRM2. Collectively, we reveal for the first time that Cyclin Y promotes lung cancer radioresistance by binding to Chk1 to activate RRM2/STAT3 signaling, indicating that targeting Cyclin Y may be a promising strategy for enhancing the efficacy of radiotherapy in the treatment of non-small cell lung cancer.

## Introduction

Lung cancer is a highly malignant neoplasm with substantial morbidity and mortality worldwide 1. Among its multiple histologic subtypes, non-small cell lung cancer (NSCLC) represents the most prevalent form 2. Contemporary therapeutic modalities for NSCLC include surgical intervention, chemotherapy, radiotherapy, targeted therapy, and immunotherapy 3. Despite the advent of diverse radiotherapy modalities that have significantly improved treatment for NSCLC patients, the clinical effectiveness of radiotherapy remains constrained by the intrinsic radioresistance of lung cancer cells 4. Consequently, there is a pressing need to identify novel targets and innovative strategies to enhance the treatment outcomes of lung cancer radiotherapy.

Cell cycle dysregulation has been involved in the proliferation, invasion, and metastasis of tumors 5, 6. Among the three key components responsible for controlling the cell cycle, namely cyclins, cyclin-dependent kinases (CDKs), and CDK inhibitors, the binding of CDKs to specific sites on cyclins is necessary for the formation of CDK-cyclin complexes, which drive cell cycle progression 7, 8. Cyclin Y, a member of the cyclin family, is involved in various biological processes, such as memory flexibility, spine plasticity, autophagy, adipogenesis, and cancer progression 9-12. The involvement of the Cyclin Y/CDK16 complex in regulating AMPK-induced autophagy has been revealed 13. Furthermore, increased expression of Cyclin Y has been shown to potentially enhance the metastatic potential of lung cancer cells by regulating cytoskeletal assembly 14. These findings suggest a plausible association between abnormal Cyclin Y expression and the development of cancer. However, the role and regulatory mechanism of Cyclin Y in lung cancer radiotherapy have yet to be elucidated.

The small subunit of ribonucleotide reductase, known as ribonucleotide reductase M2 (RRM2), plays crucial roles in nucleotide metabolism and various processes related to DNA biosynthesis, repair, and replication 15, 16. Studies have demonstrated that RRM2 is involved in the modulation of diverse signaling pathways 17-19. For example, RRM2 has been found to activate the ANXA1/AKT signaling axis, thereby modulating antitumor immune responses in renal cancer 17. Additionally, RRM2 has been shown to activate the JAK2/STAT3 signaling pathway and promote the development of retinoblastoma 18. Moreover, Chk1 inactivation was observed to result in decreased RRM2 expression in glioblastoma and neuroblastoma 20, 21. The above results indicate the potential involvement of RRM2 in the process of tumorigenesis and its potential effect on radiosensitivity.

In this study, we reveal that Cyclin Y is overexpressed and that Cyclin Y silencing suppresses lung cancer cell proliferation and enhances radiosensitivity both *in vitro* and *in vivo*. Importantly, we demonstrate that Cyclin Y interacts with Chk1 to positively modulate the expression of RRM2 and activate STAT3 signaling, ultimately promoting radioresistance in lung cancer.

## Methods and Materials

### Cell culture and plasmid transfection

All cell lines were acquired from the American Type Culture Collection and cultured in RPMI-1640 or DMEM medium supplemented with 10% FBS and 100 μg/mL penicillin/streptomycin, and subsequently incubated at 37°C under 5% CO_2_. The Myc-RRM2, pDNOR-Cyclin Y and pDNOR-Chk1 plasmids were obtained from Vigene Biosciences (Rockville, MD, USA). The pDNOR-Cyclin Y and pDNOR-Chk1 plasmids were transferred to a destination vector with SFB or MYC tag by Gateway Technology (Invitrogen, Carlsbad, CA, USA). Transfection was conducted using Lipofectamine 2000 reagent (Invitrogen) for a duration of 48 h.

### Irradiation

The cells were cultured in 6 cm flat dishes and subjected to various doses of radiation (0, 2, 4, 6, and 8 Gy) at a rate of 6 Gy/min using a Varian X-ray irradiator. After a two-week period, the number of surviving colonies was determined. In the context of the neutral comet assay and foci formation assay, cells were seeded in 24-well plates and exposed to a 6 Gy dose of radiation the following day. For the cellular radiotherapy experiments, the cells were treated for 48 h, and after 24 h of irradiation with a 6 Gy dose, the cells were harvested for protein extraction. When the average volume of xenografts in the mouse radiosensitivity experiment reached 100 mm^3^, a 10 Gy dose of local irradiation was administered.

### Antibodies and reagents

Primary antibodies specific for Cyclin Y (18042-1-AP), RRM2 (11661-1-AP), STAT3 (10253-2-AP), Myc-Tag (66004-I-Ig), Chk1 (25887-1-AP) and GAPDH (60004-1-Ig) were obtained from Proteintech (Wuhan, China). Anti-phospho-STAT3 (Tyr705) antibody (#9145) and anti-phospho-Chk1 (Ser345) antibody (#2348) were acquired from Cell Signaling Technology (Danvers, MA, USA). The anti-Cyclin Y antibody (HPA0362905) utilized for immunohistochemical (IHC) analysis was purchased from Sigma-Aldrich (St. Louis, MO, USA).

### RNA interference

Lipofectamine RNAiMAX reagent (Invitrogen) was used to transfect A549 and H1299 cells with Cyclin Y-, RRM2- and Chk1-targeting siRNAs for a duration of 48 h. The siRNA sequences were as follows:

SiCyclin Y#1: 5′-GCAAGAGUCUCUUCAUUAATT-3′,

SiCyclin Y#2: 5′-CCAUCAUCCUCCAGGACAATT-3′,

SiRRM2: 5′-CCAUCGAGUACCAUGAUAUTT-3′,

SiChk1#1: 5´-TTTGGTAAAGAATCGTGTC-3´ 22 and

SiChk1#2: 5´-GGAGAGAAGGCAAUAUCCAtt-3´ 23.

### Lentiviral transduction

Lentiviral particles carrying the Cyclin Y or control sgRNAs were generated through the transfection of HEK293T cells with the viral packaging plasmids pSPAX2 and pMD2G. After a 48-h incubation period, the supernatants containing the lentivirus were collected, filtered, and used to infect A549 cells. The cells were supplemented with 10 μg/mL polybrene and incubated for two days. Stable cell lines were selected by incubation with 2 μg/mL puromycin and were confirmed by western blotting. The specific Cyclin Y sgRNA sequences used were as follows:

SgCyclin Y#1: 5′-GUUGUUUUCUAGCAUCCUCC-3′;

SgCyclin Y#2: 5′-UUCCUUGCUAUUUGUCCUGG-3′.

### Western blotting and co-immunoprecipitation (co-IP)

The cells were lysed using NETN lysis buffer and subsequently centrifuged to collect the supernatant. The protein concentration was determined, and equal quantities of protein were denatured at 100°C for 10 min. The proteins were then separated by SDS-PAGE and transferred onto PVDF membranes. The samples were incubated overnight with primary antibodies, followed by incubation with the corresponding secondary antibodies. Bands were detected using enhanced chemiluminescence, and the densities of the bands were quantified. For exogenous interactions, samples are subjected to overnight incubation with S-beads (Novagen, Madison, WI, USA) at 4°C followed by Western blotting analysis, while for detection of endogenous binding, protein lysates are incubated with anti-Cyclin Y antibody and protein A/G agarose (Santa Cruz Biotechnology, Santa Cruz, CA, USA) overnight at 4°C for Western blotting analysis.

### RNA transcriptome sequencing (RNA-seq) analysis

The cells were transfected with scramble or Cyclin Y-targeting siRNA, and total RNA was isolated using TRIzol reagent (Takara Biotechnology, Kusatsu, Japan). The RNA was subjected to RNA-seq analysis at Novogene (Beijing, China) following quality control measures. The NovoMagic platform was utilized for conducting differential gene expression analysis. All the obtained data have been deposited in the Gene Expression Omnibus database under the assigned registration number GSE246182.

### RNA extraction and real-time quantitative PCR

An RNA extraction kit (Omega, Norcross, GA, USA) was employed to extract total RNA from cells that had been transfected with the indicated siRNAs. Reverse transcription and real-time quantitative PCR were conducted using a Vazyme kit. The relative mRNA levels of the target genes were determined using the ΔCt method, with GAPDH serving as the internal reference gene. The primer sequences utilized in this study are presented in Supplemental Table S1.

### Cell growth and colony formation assays

The cells were collected and quantified using a Countstar. For the growth assays, the cell numbers were assessed on days 1, 3, 5, and 7, with an initial seeding of 1×10^5^ cells per well. The colony formation assays involved the plating of 800 cells per well, and the visible colonies were counted after 10-14 days.

### EdU assay

EdU incorporation was evaluated by employing an EdU-594 kit (Beyotime, C0078S, Shanghai, China). The cells were incubated with 10 mM EdU at 37°C for a duration of 2 h, subsequently fixed, permeabilized with 0.3% Triton X-100 at 4°C, and subjected to a click reaction and Hoechst 33342 staining. The EdU-positive cells were captured using a fluorescence microscope and quantified.

### Clonogenic cell survival assay

A549 and H1299 cells transfected with siRNA were gathered and enumerated. Subsequently, the cells were divided into five groups according to the radiation dose (0, 2, 4, 6, or 8 Gy), and three wells per group were inoculated with 200, 400, 1000, 4000, or 8000 cells. After two weeks, the cells were immobilized, stained, and imaged. Survival curves were fitted by the single-hit multitarget model using GraphPad Prism software.

### Neutral comet assay

A comet assay kit (Trevigen, 4250-050-K, USA) was used to assess DNA damage. The cells were irradiated (6 Gy) and collected after 4 h. Subsequently, a cell suspension was prepared by mixing the cells with low melting point agarose (LMA) at a ratio of 1:10. The resulting mixture was evenly spread onto comet slides, which were subsequently subjected to lysis and electrophoresis. The slides were immersed in DNA precipitation solution for 30 min at room temperature, stained with SYBR Gold for 10 min and photographed by fluorescence microscopy.

### Immunofluorescence staining

A549 and H1299 cells were cultured on small round slides placed in 24-well plates. Following exposure to 6 Gy irradiation, the cells were fixed and permeabilized within a time frame of 4 h. Subsequently, the cells were incubated overnight with primary antibodies targeting γH2AX (Abcam, ab26350, Cambridge, MA, UK) and Rad51 (Abcam, ab133534, Cambridge, MA, UK). The following day, the cells were treated with the corresponding secondary antibodies prior to DAPI staining. The samples were imaged by confocal microscopy.

### Immunohistochemical (IHC) staining

Lung cancer tissues and paired paracancerous tissues were acquired from Shanghai Outdo Biotech (Shanghai, China). The collected nude mouse tumor tissues were fixed in 4% paraformaldehyde and subsequently employed for immunohistochemical analysis. After a 20-min incubation in 3% hydrogen peroxide at room temperature, the tissues were blocked with goat serum. Primary antibodies against Cyclin Y (1:100, Sigma, HPA0362905) and Ki-67 (1:400, Proteintech, 27309-1-AP), were incubated with the samples overnight at 4°C. The membranes were incubated with secondary antibodies at room temperature the next day. The sections were subsequently dehydrated and stained with hematoxylin. The expression of Cyclin Y and Ki-67 was assessed using the IHC score, which accounts for the intensity of cellular staining and the proportion of positive cells.

### *In vivo* xenograft mouse mode

Female BALB/c nude mice aged 4~6 weeks were randomly allocated to different groups. Sg control and Sg-Cyclin Y A549 cells were cultured to enable the inoculation of 5×10^6^ cells per nude mouse. The tumors were assessed starting approximately 10 days postinoculation and subsequently at intervals of 3 days, with the tumor volume being calculated using the formula V=(Length × Width^2^) /2. In the radiotherapy group, once the tumor volume reached 100 mm^3^, the tumor site was subjected to 10 Gy local irradiation. The animal experiments were approved by The Medical Ethics Committee of Tongji Medical College, Huazhong University of Science and Technology.

### Statistical analysis

The experiments were conducted independently and replicated three times. The data are expressed as the mean ± standard deviation (SD) unless otherwise specified. A two-tailed Student's t test was employed to assess the statistical differences between the groups, with a significance level set at P < 0.05.

## Results

### Cyclin Y is highly expressed and predicts poor prognosis in non-small cell lung cancer

**Figure 1:**
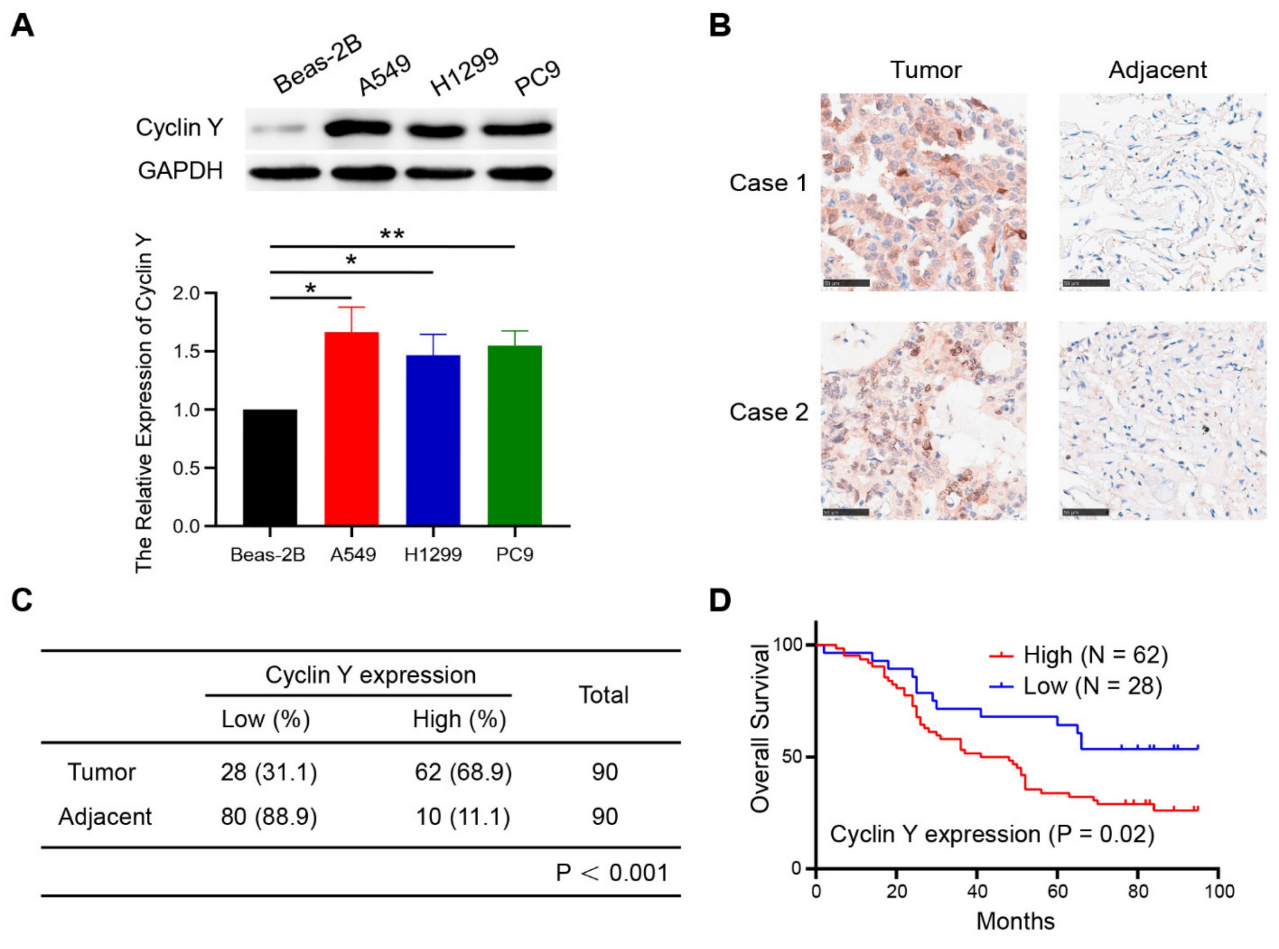
** Cyclin Y is highly expressed and predicts poor prognosis in lung cancer.** (A) Upper panel: Cyclin Y protein levels in various cell lines were measured by western blotting. Lower panel: The grayscale values of the protein bands were quantified. *P < 0.05, **P < 0.01 (n = 3). (B) Representative IHC staining images for Cyclin Y in lung cancer and adjacent lung tissues are presented. Scale bar, 100 µm. (C) Immunohistochemical staining analysis was conducted to assess Cyclin Y expression in the lung adenocarcinoma tissue microarrays. (D) Overall survival curve showing that high expression of Cyclin Y predicts lower survival in lung cancer patients.

To explore the expression of Cyclin Y in lung cancer and its potential prognostic significance, we performed western blot analysis on several lung cancer cell lines. As shown in Fig. 1A, Cyclin Y expression was upregulated in three cancer cell lines (H1299, A549, and PC9) compared with that in human bronchial epithelial Beas-2B cells. Additionally, immunohistochemical staining of lung adenocarcinoma tissue microarrays, consisting of 90 paired cancerous and paracancerous tissues, exhibited elevated levels of Cyclin Y protein expression in lung cancer tissues (Fig. 1B and C). The results of the Kaplan-Meier survival analysis demonstrated a significant association between high Cyclin Y expression and decreased overall survival in lung cancer patients (Fig. 1D). These findings suggest that Cyclin Y may serve as an oncoprotein in lung cancer.

### Knockdown of Cyclin Y inhibits tumorigenesis *in vitro* and *in vivo*

**Figure 2:**
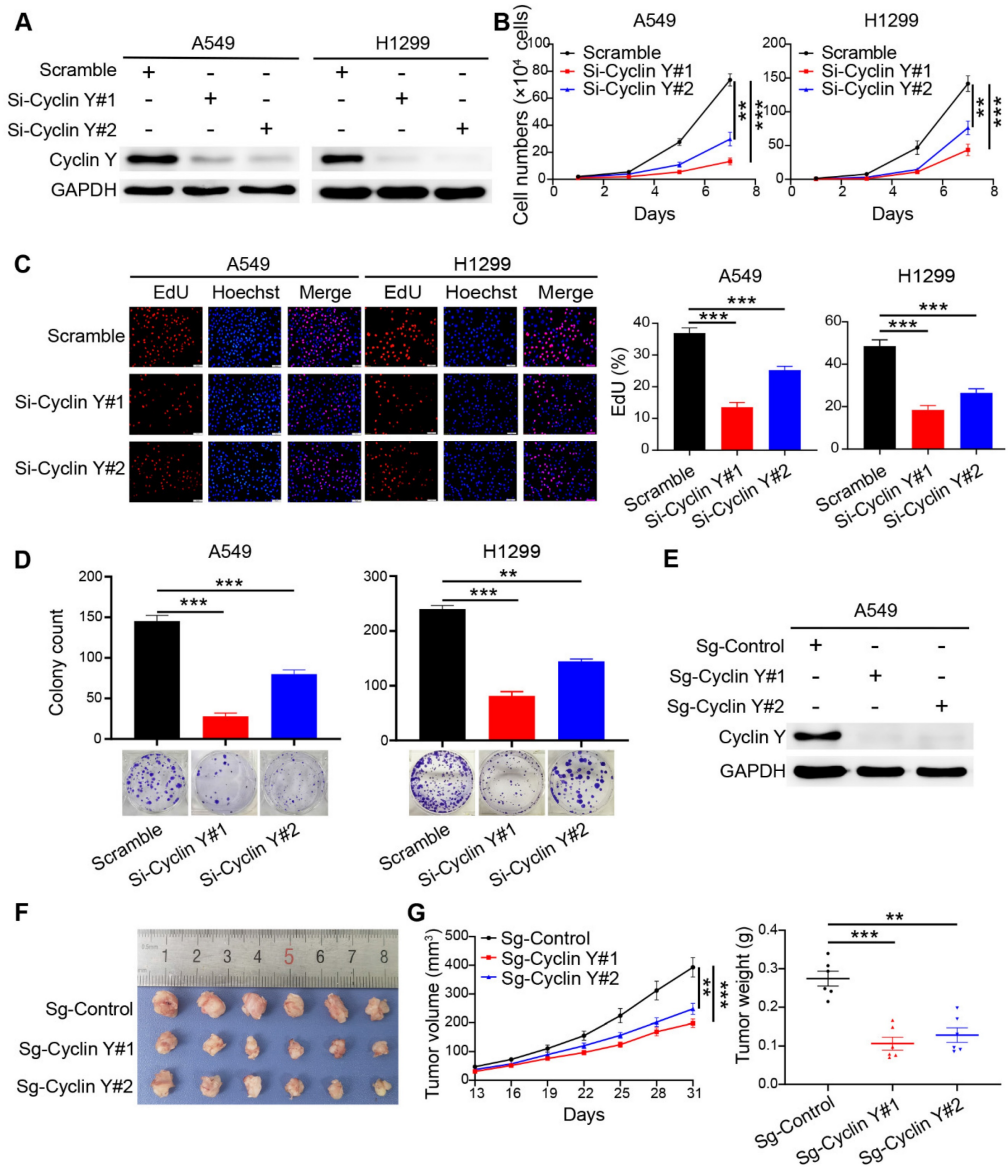
** Knockdown of Cyclin Y inhibits lung cancer cell growth and proliferation *in vitro* and *in vivo*.** (A) Two specific siRNAs were utilized to effectively suppress the expression of Cyclin Y in A549 and H1299 cells (n = 3). (B) The siRNAs were transfected into six-well plates and cell counts were recorded every two days. ***P < 0.001 (n = 3). (C) Left panel: EdU incorporation assays were used to assess cell proliferation. Right panel: proportion of EdU-positive cells in each group. Scale bar, 50 µm. ***P < 0.001 (n = 3). (D) Transfected cells were seeded in six-well plates and cultured for two weeks. The number of colonies consisting of more than 50 cells was quantified. **P < 0.01, ***P < 0.001 (n = 3). (E) Two Cyclin Y-targeting sgRNAs were employed to effectively knock down Cyclin Y in A549 cells (n = 3). (F) Image of subcutaneous xenograft tumors (six mice per group). (G) Tumor volumes and wet weights were calculated every three days for the xenograft tumors (six mice per group). The data are presented as the mean ± SEMs. **P < 0.01, ***P < 0.001.

Based on the observed elevated expression of Cyclin Y and its potential prognostic implications, we hypothesized that Cyclin Y plays an oncogenic role in lung cancer. To confirm this hypothesis, we utilized RNA interference to effectively silence Cyclin Y in the A549 and H1299 cell lines, which reduced Cyclin Y expression by more than 90% (Fig. 2A). The knockdown of Cyclin Y resulted in significant inhibition of lung cancer cell growth and proliferation (Fig. 2B and D). Additionally, the decrease in the proportion of EdU-positive cells following Cyclin Y depletion suggested the suppression of cell proliferation (Fig. 2C). Conversely, the overexpression of Cyclin Y facilitated the growth and proliferation of lung cancer cells (Fig. S1). To further validate these effects *in vivo*, we established Cyclin Y stable knockdown cells and found a reduction in both tumor size and weight in nude mice injected with Cyclin Y knockdown cells compared with those injected with control cells (Fig. 2E-G). Thus, these findings provide evidence that Cyclin Y depletion indeed inhibits tumorigenesis both* in vitro* and *in vivo*.

### Cyclin Y silencing increases DNA damage and impairs DNA repair in lung cancer cells

**Figure 3:**
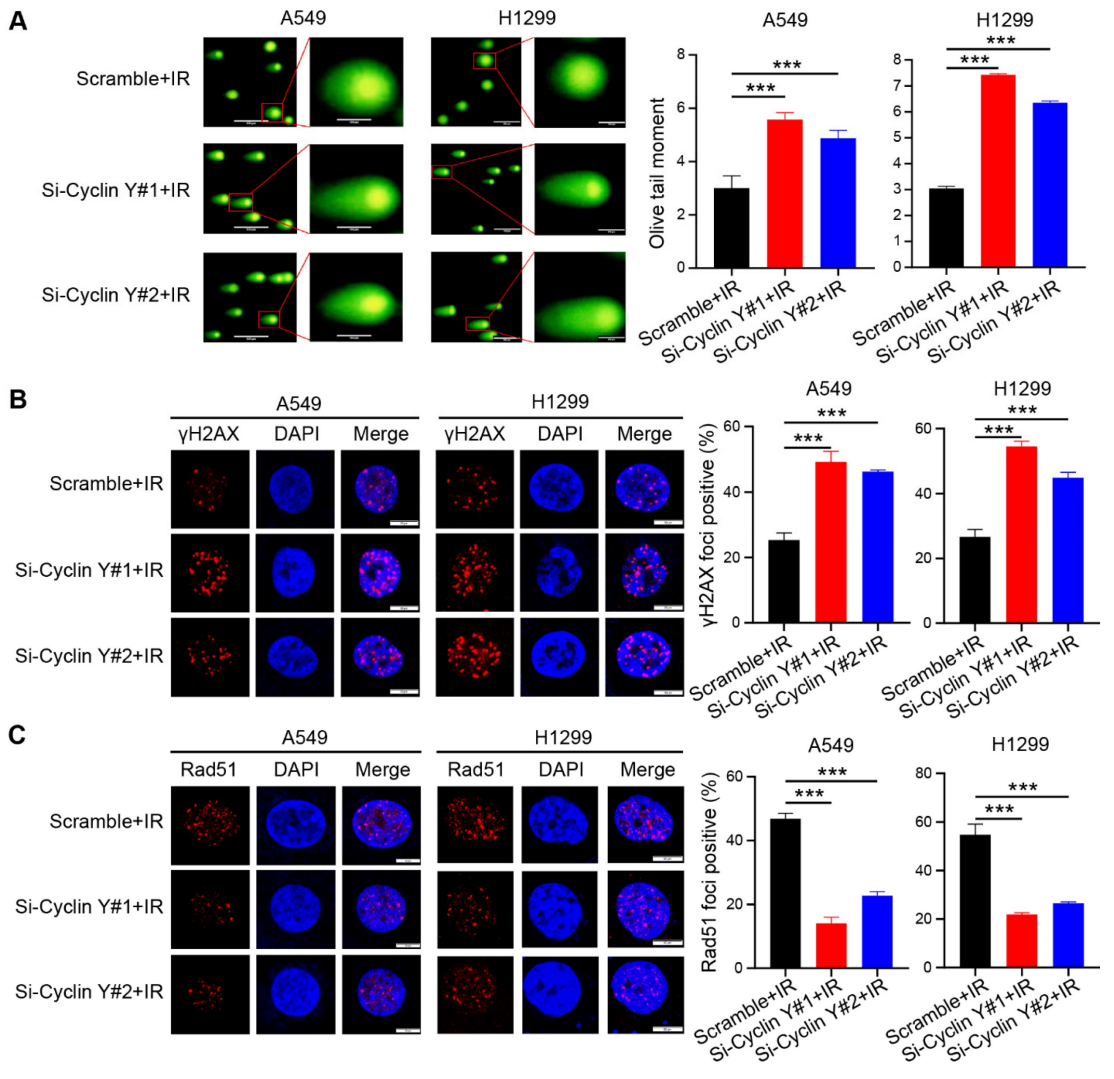
** Cyclin Y silencing increases DNA damage and impairs DNA repair in lung cancer cells.** (A) The Olive tail moment was calculated and graphed for each group. Scale bar, 100 µm. ***P < 0.001 (n = 3). (B) Left panel: Cells transfected with the indicated siRNAs were irradiated and harvested after 4 h, followed by photography. Right panel: A quantitative analysis is presented. Scale bar, 50 µm. ***P < 0.001 (n = 3). (C) Left panel: Cells were transfected with the indicated siRNAs, irradiated, harvested, and analyzed for Rad51 foci. Right panel: A quantitative analysis is presented. Scale bar, 50 µm. ***P < 0.001 (n = 3).

Radioresistance, which is known to be a significant factor contributing to unfavorable outcomes in cancer treatment, often arises as a result of abnormal DNA damage repair in tumor cells 24, 25. To investigate the potential impact of Cyclin Y on DNA damage repair, we conducted a neutral comet assay and observed an increase in the comet tail moment in cells with Cyclin Y knockdown following irradiation (Fig. 3A). Previous research has demonstrated that γH2AX and Rad51 foci can serve as reliable indicators of the DNA damage response (DDR) after irradiation 26. In line with the findings from the comet assay, the number of γH2AX foci, a well-established marker of DNA damage, increased similarly following irradiation. (Fig. 3B). Moreover, a decrease in Rad51 foci was observed in Cyclin Y-depleted cells (Fig. 3C), suggesting that Cyclin Y knockdown hampers the ability to repair DNA damage. Notably, depletion of Cyclin Y did not lead to significant changes in the cell cycle distribution or DNA damage levels in the absence of irradiation in lung cancer cells (Fig. S2). Taken together, these results demonstrate that the depletion of Cyclin Y in lung cancer cells results in an increase in residual DNA damage and an impairment in DNA damage repair following irradiation.

### Cyclin Y depletion increases radiosensitivity in lung cancer *in vitro* and *in vivo*

**Figure 4:**
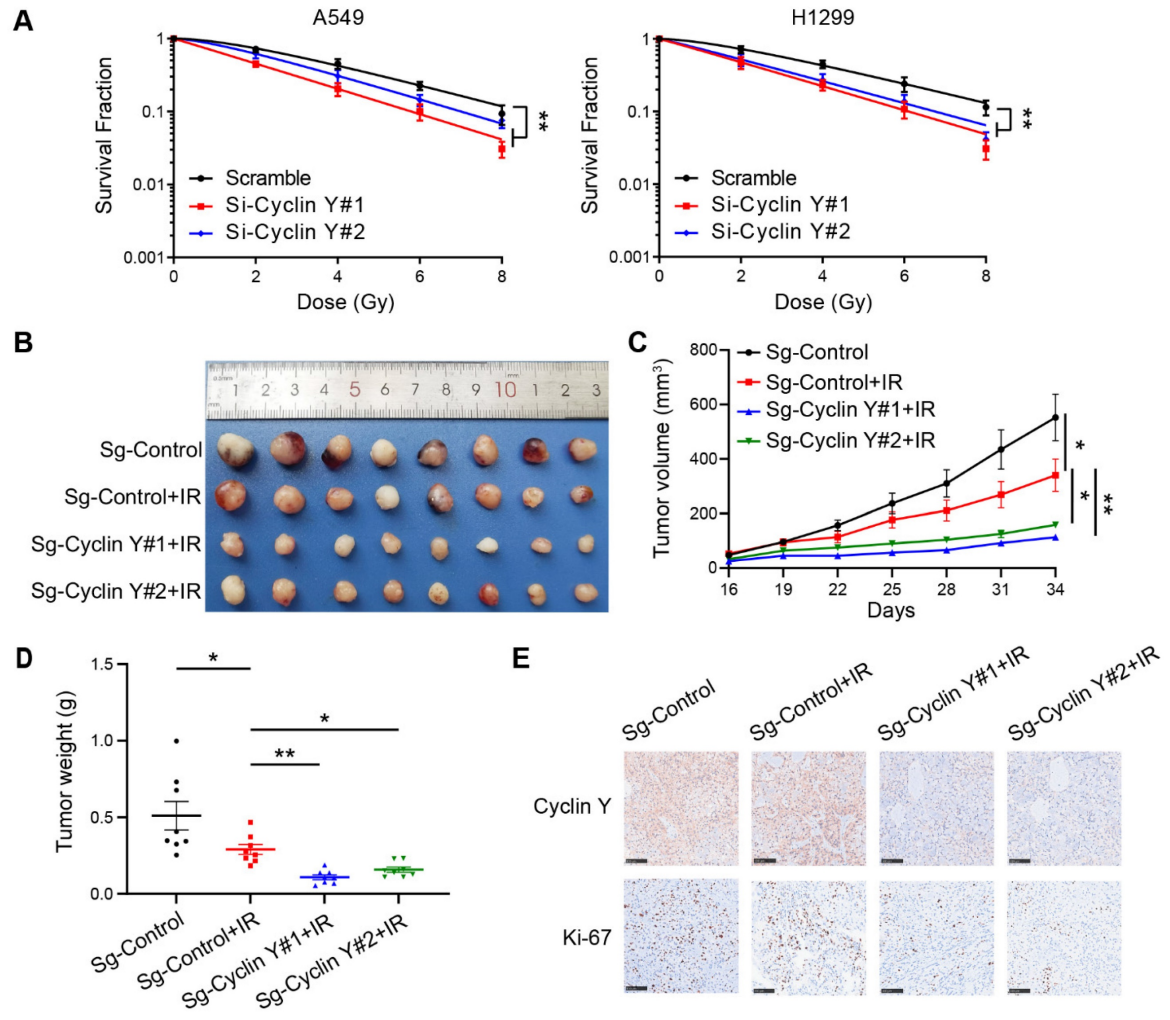
**Cyclin Y depletion enhances radiosensitivity in lung cancer *in vitro* and *in vivo*.** (A) The depletion of Cyclin Y using siRNAs resulted in increased sensitivity to radiation *in vitro*. The number of colonies was quantified after 2 weeks. **P < 0.01 (n = 3). (B) Representative image of xenograft tumors in the four groups. A total of 5×10^6^ A549 cells were injected subcutaneously into the right posterior limb of each mouse. When the tumor volume reached an average calculated volume of 100 mm^3^, the mice were exposed to a radiation dose of 10 Gy (eight mice/group). (C) Growth curves for each of the four experimental groups are presented. Tumor size was measured using calipers every three days. The data are plotted as the mean tumor volumes ± SEMs. *P < 0.05, **P < 0.01, ***P < 0.001. (D) The tumor wet weights of the four groups were measured (eight mice/group). (E) Immunohistochemistry (IHC) images of xenograft tumors were obtained to assess the expression of Cyclin Y and Ki-67. Scale bar, 100 µm.

Given the observed impact of Cyclin Y knockdown on DNA damage repair, it is hypothesized that Cyclin Y may be associated with radiosensitivity. Using clonogenic survival assays, we demonstrated that depletion of Cyclin Y resulted in increased radiosensitivity in lung cancer cell lines (Fig. 4A). To further validate these findings* in vivo*, xenograft tumor assays were conducted in nude mice. As shown in Fig. 4B-D, the tumor size and weight exhibited a reduction following irradiation (10 Gy), indicating the efficacy of the radiation treatment. Furthermore, after the irradiation of both cohorts of mice, the sg-Cyclin Y groups exhibited a reduction in tumor size and weight in comparison to the sg-control groups (Fig. 4B-D). This radiosensitization effect was also observed when Cyclin Y depletion was combined with different low doses of radiation (2 Gy or 4 Gy) (Fig. S3). Furthermore, the immunohistochemistry (IHC) results revealed diminished levels of Ki67 protein in the sg-Cyclin Y group subjected to irradiation (Fig. 4E). These data indicate that the downregulation of Cyclin Y expression enhances lung cancer radiosensitivity both *in vitro* and *in vivo*.

### Targeting Cyclin Y inhibits STAT3 activation partially through the downregulation of RRM2

**Figure 5:**
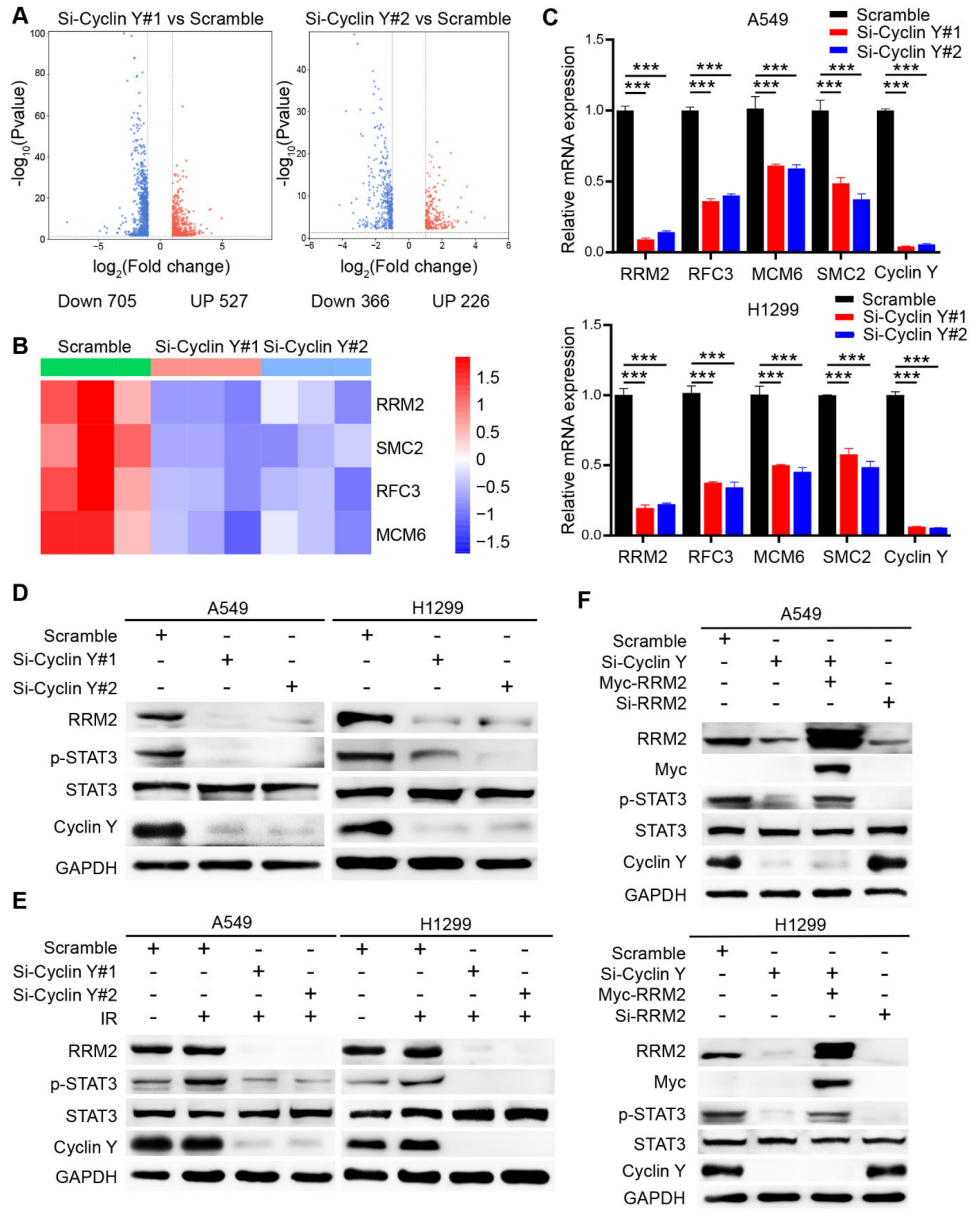
**Knockdown of Cyclin Y decreases RRM2 expression and blocks STAT3 phosphorylation in lung cancer cells.** (A) The differences in gene expression between Cyclin Y-depleted A549 cells and control cells are visually represented in volcano plots. (B) The alterations in the expression of representative genes associated with DNA damage repair are presented in a heatmap. (C) The levels of the indicated mRNAs were quantitatively measured using real-time quantitative PCR, ***P < 0.001 (n = 3). (D, E) The protein expression of RRM2 and p-STAT3 was decreased in Cyclin Y-depleted lung cancer cells under normal conditions and in response to DNA damage (n = 3). (F) A549 and H1299 cells were transfected with specific siRNAs and plasmids and subsequently collected for western blot analysis (n = 3).

To elucidate the mechanisms underlying the regulatory role of Cyclin Y in lung cancer, we conducted an RNA-seq analysis utilizing A549 cells that were transfected with control or Cyclin Y siRNAs and revealed numerous downstream genes with altered expression patterns (Fig. 5A). Notably, a cluster of four genes, namely RRM2, SMC2, RFC3 and MCM6, which have been previously associated with DNA damage repair 21, 27-29, exhibited significant changes in cells depleted of Cyclin Y (Fig. 5B). These observations were further validated through RT-qPCR, which confirmed the downregulation of the mRNA levels of these four genes, with RRM2 displaying the most pronounced decrease (Fig. 5C). Consistent with this notion, the reduction in Cyclin Y resulted in a decrease in the protein level of RRM2 both under normal circumstances and in the presence of DNA damage after irradiation (Fig. 5D and E). A previous study indicated that RRM2 plays a role in regulating the activation of the transcription factor STAT3 18. We found that knockdown of Cyclin Y or RRM2 significantly inhibited the phosphorylation of STAT3, regardless of the presence or absence of DNA damage (Fig. 5D-F). Importantly, the reduced phosphorylation of STAT3 was partially restored when exogenous RRM2 was introduced into Cyclin Y depleted cells (Fig. 5F). These findings indicate that Cyclin Y modulates the activation of STAT3 through RRM2 in lung cancer.

### Cyclin Y interacts with Chk1 to activate RRM2/STAT3 signaling in lung cancer

**Figure 6:**
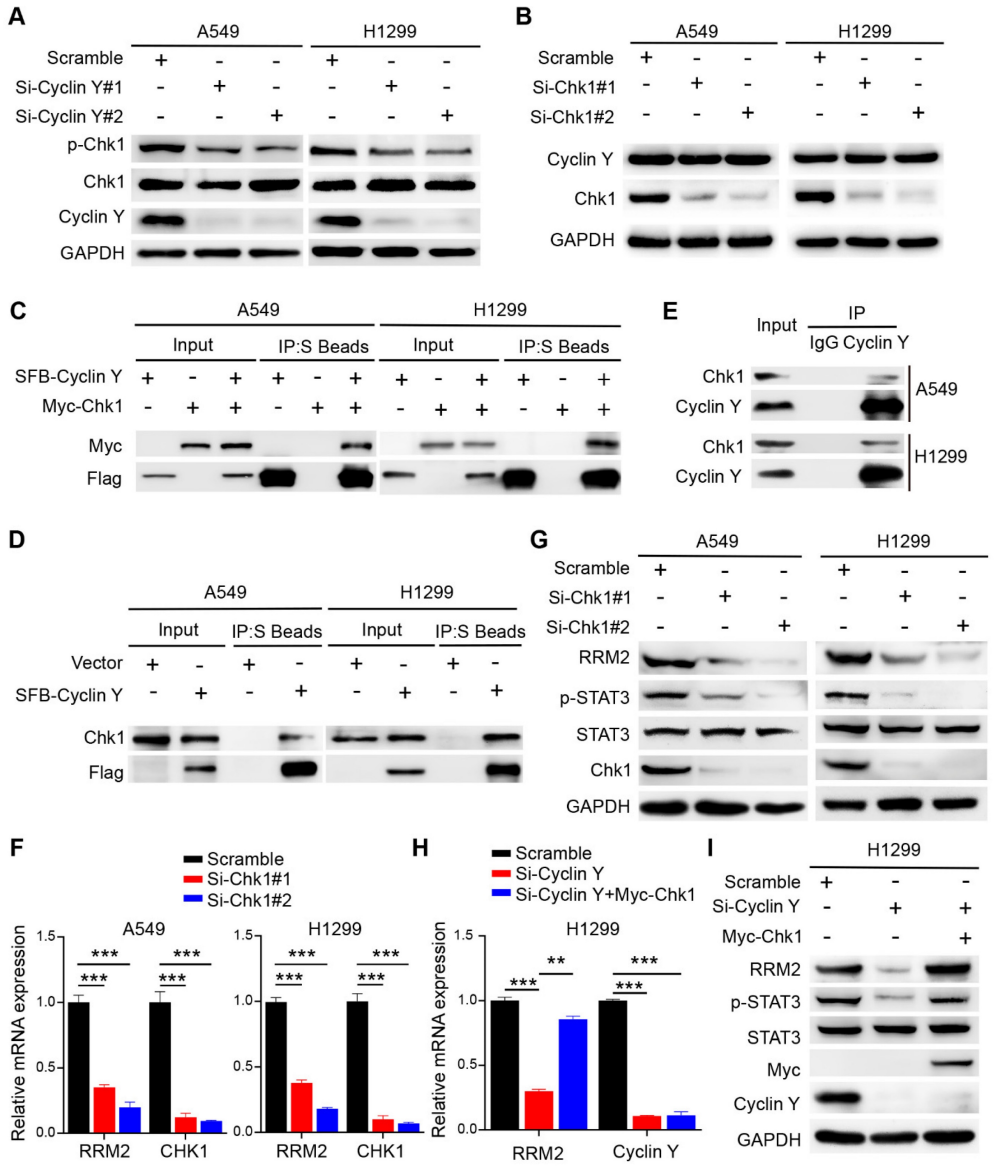
**Cyclin Y interacts with Chk1 to activate RRM2/STAT3 signaling in lung cancer.** (A) A549 and H1299 cells were transfected with the indicated siRNAs and harvested for western blot analysis (n = 3). (B) A549 and H1299 cells were transfected with targeting-Chk1 siRNAs and harvested for western blot analysis (n = 3). (C) A549 and H1299 cells transfected with SFB-Cyclin Y or Myc-Chk1 plasmids were harvested and co-immunoprecipitated with S-protein agarose (n = 3). (D) A549 and H1299 cells transfected with the SFB-Cyclin Y plasmid were harvested and co-immunoprecipitated with S-protein agarose (n = 3). (E) Endogenous Cyclin Y binds to endogenous Chk1 in A549 and H1299 cells (n = 3). (F) A549 and H1299 cells transfected with targeting-Chk1 siRNAs were harvested for RNA extraction. The levels of RRM2 and Chk1 mRNAs were quantitatively measured using real-time quantitative PCR, ***P < 0.001 (n = 3). (G) A549 and H1299 cells transfected with targeting-Chk1 siRNAs were harvested for western blot analysis (n = 3). (H) H1299 cells were transfected with the indicated siRNAs and plasmids and then harvested for mRNA analysis. **P < 0.01, ***P < 0.001 (n = 3). (I) H1299 cells were transfected with the indicated siRNAs and plasmids and then harvested for western blot analysis (n = 3).

Previous studies have established the regulatory role of Chk1 in RRM2 expression and its involvement in DNA damage response 20, 21. Consequently we speculated that Chk1 may play a role in the regulation of RRM2 by Cyclin Y. As expected, Cyclin Y depletion downregulated the phosphorylation of Chk1, while the total levels of Chk1 were not altered (Fig. 6A). Notably, depletion of Chk1 did not alter the protein expression of Cyclin Y (Fig. 6B). Moreover, we showed that exogenously expressed Cyclin Y formed a complex with exogenous or endogenous Chk1 in lung cancer cells (Fig. 6C and D). The endogenous interaction of Cyclin Y with Chk1 was also observed (Fig. 6E). These results confirm that Cyclin Y interacts with Chk1 in lung cancer cells.

Consistent with our hypothesis, the depletion of Chk1 led to a decrease in both the mRNA and protein levels of RRM2, accompanied by the concurrent inhibition of STAT3 phosphorylation (Fig. 6F and G). To explore whether Cyclin Y controls the expression of RRM2 via Chk1, rescue experiments were performed. As shown in Fig. 6H and I, the overexpression of Chk1 partially reversed the decreases in the mRNA and protein levels of RRM2 as well as the activation of STAT3 in Cyclin Y-depleted cells. These findings indicate that Cyclin Y controls the activation of RRM2/STAT3 signaling in a Chk1-dependent manner in lung cancer cells.

### Cyclin Y exerts its biological functions in lung cancer cells partially through the positive regulation of RRM2

**Figure 7:**
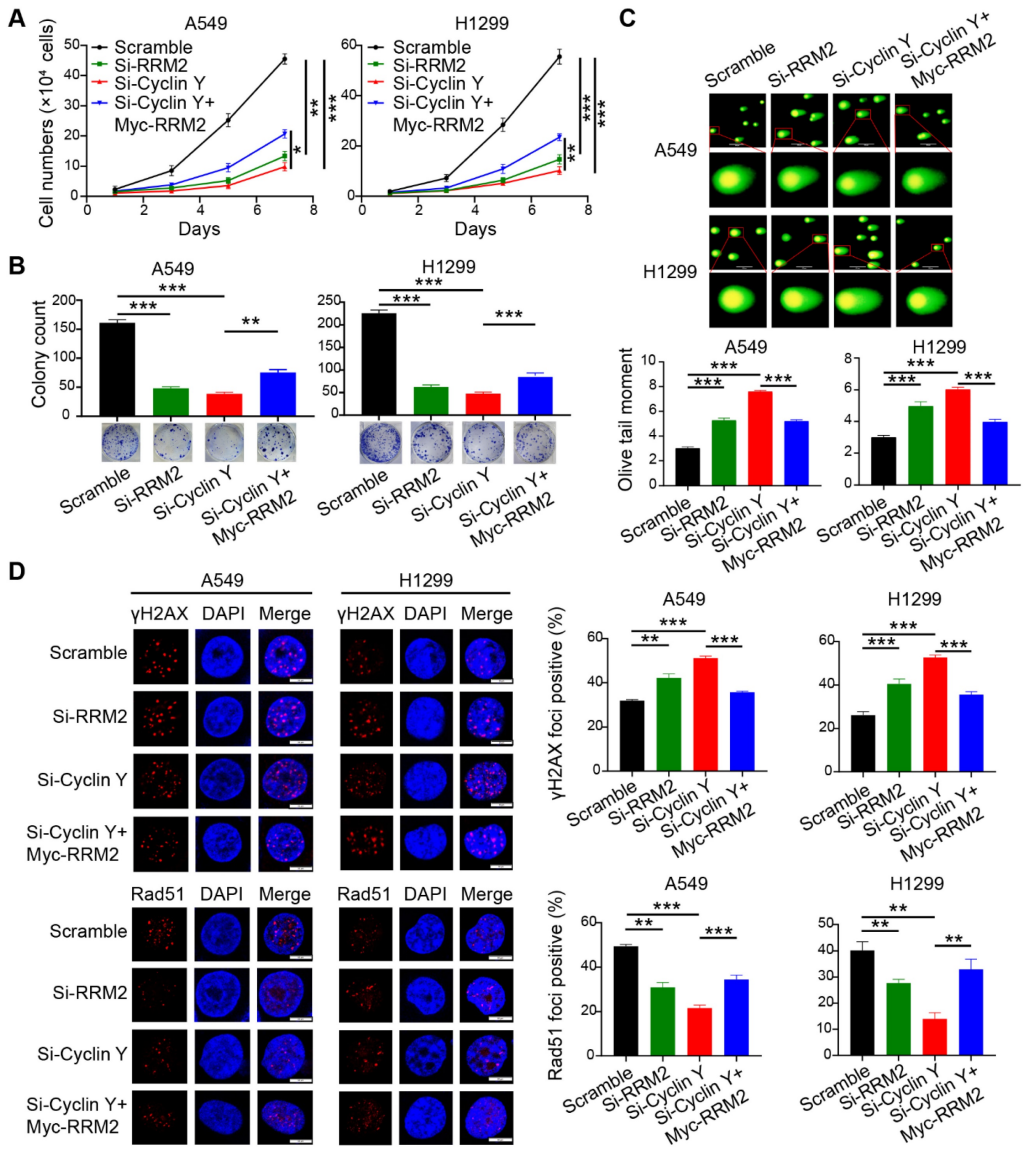
** Cyclin Y exerts its biological functions in lung cancer cells partially by positively regulating RRM2.** (A) A549 and H1299 cells were transfected with specific siRNAs and plasmids and then seeded in six-well plates. Cell counting was performed every two days. **P < 0.01, ***P < 0.001 (n = 3). (B) A549 and H1299 cells were seeded and cultured for 14 days, followed by staining and counting of the colonies. **P < 0.01, ***P < 0.001 (n = 3). (C) Neutral comet assays were conducted to assess the extent of DNA damage following irradiation in each experimental group. Scale bar, 100 µm. ***P < 0.001 (n = 3). (D) DNA damage and repair were evaluated through γH2AX and Rad51 foci formation assays. Representative immunofluorescence images and statistical diagrams are illustrated. Scale bar, 50 µm. **P < 0.01, ***P < 0.001 (n = 3).

To investigate the involvement of RRM2 in the effect of Cyclin Y knockdown, a series of rescue experiments were conducted in A549 and H1299 cells. Specifically, siRNAs targeting Cyclin Y and RRM2 were transfected simultaneously and separately, followed by the transfection of RRM2 plasmids containing the Myc tag into Cyclin Y knockdown cells after 24 h. The results demonstrated that the inhibition of RRM2 led to a decrease in lung cancer cell growth and proliferation (Fig. 7A and B). Conversely, RRM2 overexpression partially reversed the inhibitory effects on cell growth and proliferation induced by Cyclin Y depletion in lung cancer cells (Fig. 7A and B). Furthermore, the Olive tail moment significantly increased in cells with Cyclin Y knockdown, which was partially mitigated by RRM2 overexpression (Fig. 7C). Similarly, the reintroduction of RRM2 partially reversed the elevation of γH2AX foci and the reduction in Rad51 foci formation induced by Cyclin Y knockdown (Fig. 7D and E). These findings suggest that Cyclin Y modulates the biological effects in lung cancer cells, at least in part, through the regulation of RRM2.

## Discussion

In this study, we present evidence demonstrating that Cyclin Y is overproduced and that Cyclin Y silencing impairs tumorigenesis and radioresistance in non-small cell lung cancer *in vitro* and *in vivo*. Furthermore, we identify RRM2 as a crucial downstream effector of Cyclin Y involved in the biological processes associated with Cyclin Y. These findings suggest that targeting Cyclin Y could serve as a promising approach for enhancing the efficacy of radiotherapy in lung cancer treatment.

Cyclin Y, a crucial molecule implicated in the regulation of the cell cycle, is involved not only in normal biological processes but also in the pathogenesis and progression of tumors 12, 30, 31. For instance, heightened expression of Cyclin Y notably enhances cell proliferation and migration in hepatocellular carcinoma 31. In our study, we found that Cyclin Y exhibits elevated expression levels in lung cancer tissues, which is strongly correlated with an unfavorable prognosis. Furthermore, we demonstrated that Cyclin Y depletion suppresses lung cancer progression both *in vitro* and *in vivo*. Importantly, we revealed the previously unknown role of Cyclin Y in promoting radioresistance in lung cancer. Suppression of Cyclin Y was found to induce DNA damage and impair DNA repair, consequently augmenting the radiosensitivity of lung cancer cells. Our findings provide novel insights into the biological significance of Cyclin Y in lung cancer radioresistance.

The involvement of RRM2 in nucleotide metabolism and its impact on tumor progression have been extensively investigated 16. It has been documented that the expression of RRM2 can be regulated at multiple levels 32-34. For instance, E2F transcription factors have been observed to upregulate RRM2 in pancreatic cancer, while BRCA1 has been demonstrated to transcriptionally activate RRM2 in glioblastoma. Furthermore, the deubiquitinase USP12 has been discovered to positively modulate the protein abundance of RRM2 through deubiquitination 34. Using RNA-seq technology, we show for the first time that Cyclin Y acts as a new upstream regulator of RRM2, which modulates its mRNA and protein levels under normal conditions and in response to DNA damage. In addition, this study reveals that Cyclin Y positively controls the activation of STAT3 in an RRM2-dependent manner. Importantly, we reveal that Cyclin Y interacts with Chk1, which subsequently positively regulates both the mRNA and protein expression levels of RRM2, thereby influencing the activation of STAT3. It has been well established that in the presence of DNA damage, Chk1 is predominantly activated by ATR kinase 35, 36. Therefore, we propose that Cyclin Y may facilitate the activation of Chk1 by modulating ATR kinase activity, which warrants further investigation. Notably, we demonstrate that the biological effects caused by Cyclin Y knockdown can be partially rescued by RRM2, highlighting the significance of the Cyclin Y/RRM2 axis in the regulation of lung cancer progression and radiosensitivity.

In summary, our study is the first to reveal the role of Cyclin Y in lung cancer radioresistance. Cyclin Y interacts with Chk1, thereby activating the RRM2/STAT3 signaling pathway and promoting radioresistance in lung cancer. Our research not only provides partial insight into the mechanism by which Cyclin Y functions, but also suggests that targeting Cyclin Y represents a promising approach for enhancing the sensitivity of non-small cell lung cancer to radiotherapy.

## Supplementary Material

Supplementary figures and table.

## Abbreviations

NSCLC: non-small cell lung cancer
CDKs: cyclin-dependent kinases
RRM2: ribonucleotide reductase M2
Chk1: checkpoint kinase 1
STAT3: signal transducer and activator of transcription 3
co-IP: co-immunoprecipitation
DDR: DNA damage response
ATR: ataxia telangiectasia mutated and Rad3-related
