# Supplementary material for: Cyclin Y interacts with Chk1 to activate RRM2/STAT3 signaling and promotes radioresistance in non-small cell lung cancer
Source: Int J Biol Sci. 2025 Feb 18;21(5):1999–2011. doi: 10.7150/ijbs.106925 (PMC11900816; doi:10.7150/ijbs.106925)
Supplement: Supplementary file 1 — Supplementary figures and table. [file ijbsv21p1999s1.pdf]

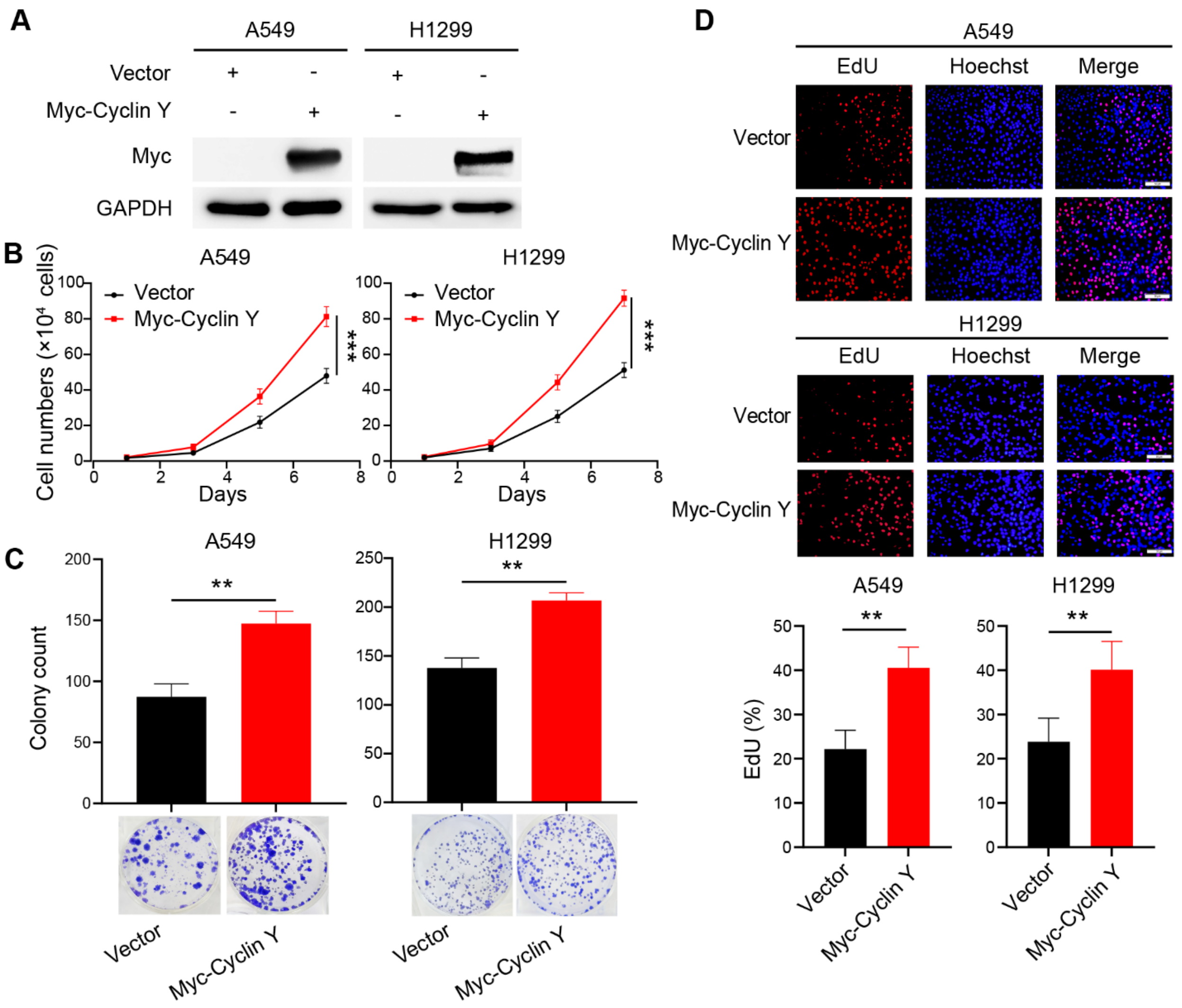

Fig. S1. The overexpression of Cyclin Y facilitates lung cancer cell growth and proliferation *in vitro*. (A) A549 and H1299 cells were transfected with the Myc-Cyclin Y plasmid, and samples were collected and analyzed by Western blotting (n = 3). (B) The Myc-Cyclin Y plasmid was transfected into six-well plates and cell counts were recorded every two days. *** $P < 0.001$ (n = 3). (C) Transfected cells were seeded in six-well plates and cultured for two weeks. The number of colonies consisting of more than 50 cells was quantified. ** $P < 0.01$ (n = 3). (D) EdU incorporation assays were used to assess cell proliferation. Scale bar, 50 μm. ** $P < 0.01$ (n = 3).

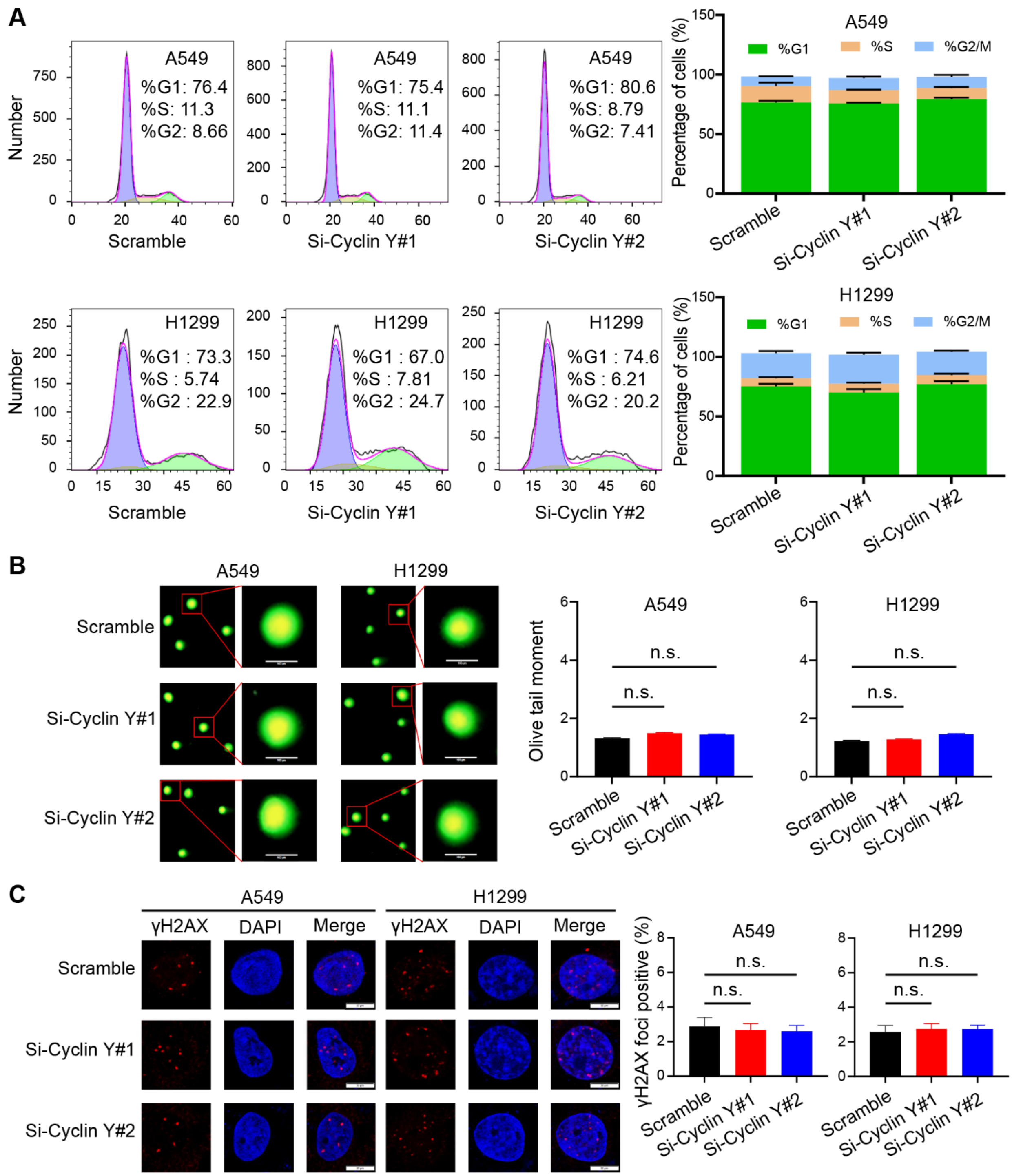

Fig. S2. Knockdown of Cyclin Y does not alter the cell cycle distribution or DNA damage levels in the absence of irradiation. (A) Cells were collected 48 h after transfection with the indicated siRNAs and analyzed by flow cytometry (n = 3). (B) The Olive tail moment was calculated and

graphed for each group (n = 3). Scale bar, 100 μm. n. s. indicates no statistically significant difference (P > 0.05). (C) Representative immunostaining images of γH2AX foci (n = 3). Scale bar, 50 μm.

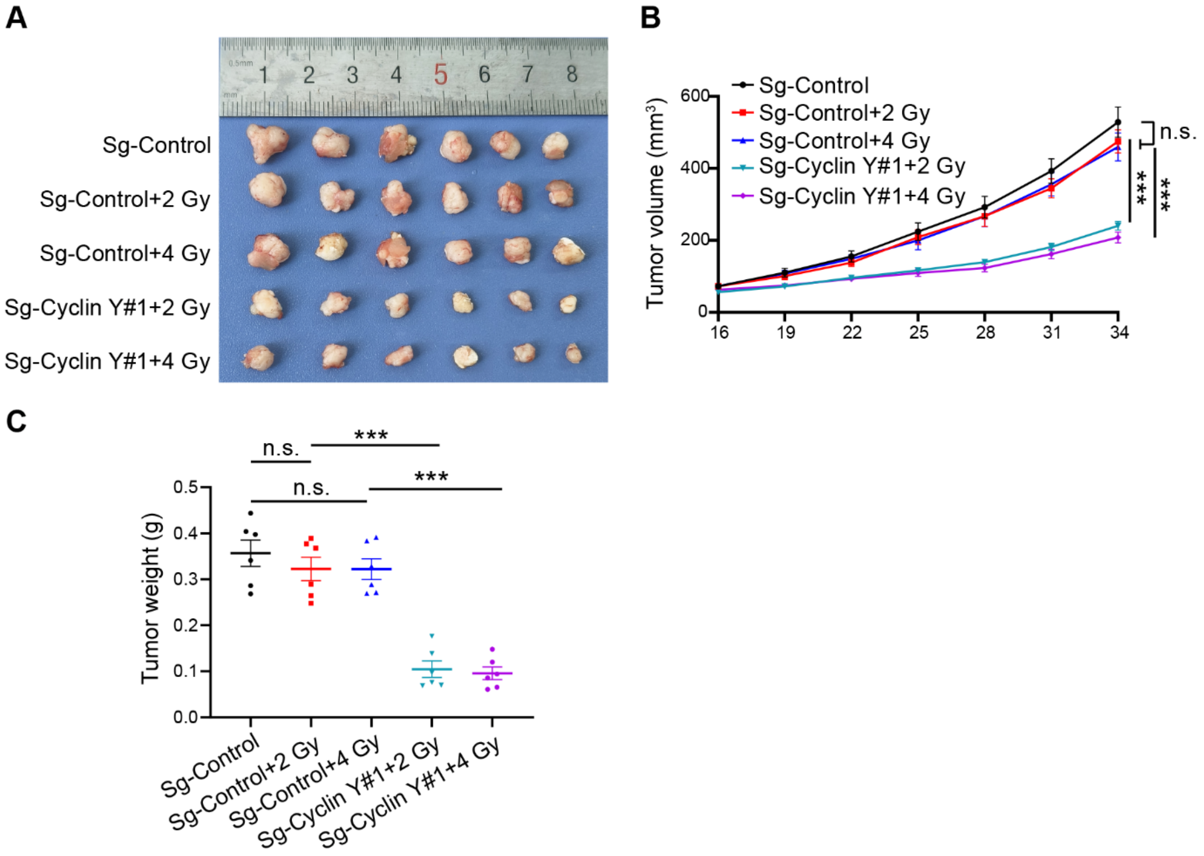

Fig. S3. Cyclin Y depletion increases radiosensitivity in lung cancer *in vivo*. (A) Representative image of xenograft tumors in the five groups. When the tumor volume reached an average calculated volume of 100 mm^3, the mice were exposed to a radiation dose of 2 Gy or 4 Gy (six mice/group). (B) The growth curves for each group are presented. The data are plotted as the mean tumor volumes ± SEMs. *** $P < 0.001$ (six mice/group). (C) The wet weights of the tumors in the five groups were measured (six mice/group). *** $P < 0.001$ (six mice/group).

Genes	Sequences (5'--3')
RRM2	F: GTGGAGCGATTTAGCCAAGAA<br>R: CACAAGGCATCGTTTCAATGG
MCM6	F: GAGGAACTGATTCGTCCTGAGA<br>R: CAAGGCCCGACACAGGTAAG
RFC3	F: ATGCCTTGCGAAGAACCA<br>R: GAACCGCCAAGCACCTAC
SMC2	F: ACAGTAACAAGGCAGGTG<br>R: ATGATGAGAAAGTGAGGGT
CHK1	F: GACAGAATAGAGCCAGACA<br>R: TTATCCCTTTCATCCAAC
Cyclin Y	F: GTTCTCCCGGTCGCTGAT<br>R: CTCGTGCTGCGTGTCGTC
GAPDH	F: GAGTCAACGGATTTGGTCGT<br>R: GACAAGCTTCCCGTTCTCAG

F, forward primer; R, reverse primer

Supplemental Table S1. Sequences of primers used for real-time quantitative PCR
